# Language impairments in Alzheimer´s disease: What changes can be found between mild and moderate stages of the disease?

**DOI:** 10.1016/j.clinsp.2024.100412

**Published:** 2024-06-19

**Authors:** Karin Zazo Ortiz, Juliana Onofre De Lira, Thais Soares Ciariancullo Minett, Paulo Henrique Ferreira Bertolucci

**Affiliations:** a Department of Speech, Language and Hearing Sciences, Universidade Federal de São Paulo, São Paulo, SP, Brazil; b Universidade de Brasília, Cinelândia Campus, Brasília, DF, Brazil; c Department of Radiology, University of Cambridge, Cambridge, UK; d Department of Neurology and Neurosurgery, Universidade Federal de São Paulo, São Paulo, SP, Brazil

**Keywords:** Dementia, Alzheimer's Disease, Language

## Abstract

**Objective:**

To investigate how language deteriorates over the Alzheimer's Disease course.

**Methods:**

A cross-sectional, observational study was carried out. 35 patients diagnosed with dementia due to AD using the NINCDS-ARDRA criteria and undergoing treatment for AD with a therapeutic dose of acetylcholinesterase inhibitors were assessed by the Boston Diagnostic Aphasia Examination (BDAE). The sample comprised 15 patients with mild AD (MMSE > 23, CDR = 0 or 0.5‒1.0) and 20 patients with moderate AD (MMSE = 13‒23, CDR = 2). The results for the 2 groups on all language tasks were compared.

**Results:**

A statistically significant difference was found between the mild and moderate AD groups for total score on the BDAE (95% CI 47.10‒114.08, *t* = 5.0, DF = 21, p = 0.000*), as well as on several tasks involving oral and writing comprehension, language oral expression and writing.

**Conclusion:**

The study results showed major changes in the moderate stage. Also, the decline in language performance correlated with the worsening of dementia syndrome, independently of sociodemographic variables.

## Highlights

• There were linguistic tasks more preserved than others during the AD's progression.
• Language is more globally affected in patients with less than 17 points on the MMSE.
• The language profile can guide strategies to maintain communication.

## Introduction

Alzheimer's Disease (AD) is the most common cause of dementia. By 2050, the number of people aged 65 and older with Alzheimer's dementia is projected to reach 12.7 million.^1^ The initial symptom of dementia in AD is memory decline, together with impairment of one or more cognitive functions, such as executive functions, visuospatial abilities, praxis, attention, and language. Patients with Alzheimer's Disease (AD) show early episodic memory impairments. Such deficits reflect specific impairments affecting one or several stages of encoding, storage, and retrieval processes.^2^ Linguistic changes can progress heterogeneously among individuals affected by the disease.^3^ Recent theories point out that even in normal elderly there are changes in performing some linguistic tasks compared with adults, and some linguistic tasks seem to demand more cognitive processing than others, so it is possible that language deterioration is not homogeneous.^4^,^5^ Given the complexity of language, the impact of disease progression on each type of linguistic processing should be monitored, since there is a need for interventions that focus on disease management.^6^ The changes in language commonly associated with the different stages of AD dementia have been the focus of many investigations.^6^ However, together with the variability in impairment of linguistic functions with disease progression,^3^ major sociodemographic disparities in developing countries can hamper accurate analysis of the impact of dementia progression on language deterioration. This occurs because, even in healthy individuals, age and education are factors that can moderate cognitive performance. With regard to language, many previous studies have confirmed that education influences linguistic performance.^5^,^7^ Similarly, aging is a factor that can impact cognitive functions in several ways, and aging effects on language have also been described.^8^ Language is a highly complex cognitive function and numerous different processes are involved in oral comprehension, oral production tasks, writing/reading comprehension, and writing. This wide range of components corroborates the hypothesis that disease progression impacts the multiple processes involved in the different language activities in a non-uniform manner. Language processing involves timely access to and retrieval of language representations. These operations are supported by cognitive abilities such as short-term memory, working memory and executive functions.^4^ Then, it is possible that linguistic tasks that demand more working memory and short memory could be more affected considering that memory is the most affected cognitive function in AD patients. Thus, comparing the performance of patients with mild and moderate AD on language tasks can further the knowledge of how disease progression impacts language. Consequently, this information can help in selecting the most effective stimulation and/or compensatory strategies for preserving communication.

Therefore, the objective of the present study was to compare the performance of individuals with mild and moderate AD on language tasks, controlling for factors such as age and education, and elucidate how disease progression impacts language.

## Materials and methods

A cross-sectional, observational study was conducted at the outpatient clinic of the Behavioral Neurology Division and at the Department of Speech, Language and Hearing Sciences of the Universidade Federal de São Paulo. The study was approved by the local research ethics committee (Permit nº 1606/03). After the participants had received full information about the study, written informed consent was obtained from all enrolled subjects.

### Study sample

The sample comprised individuals with AD. The general inclusion criteria were as follows: age ≥ 60 years; no history of alcoholism or drug use; no use of psychotropic medications, except for atypical neuroleptics; and an absence of visual or auditory impairment that might affect the outcomes of the cognitive tests. Thirty-five patients had dementia due to AD (15 mild stage and 20 moderate stage) according to the clinical criteria of the National Institute of Neurological and Communicative Disorders and Stroke – Alzheimer's Disease and Related Disorders Association (NINCDS-ADRDA) Work Group.^9^ This sample was recruited by convenience at the outpatient clinic of the Behavioral Neurology Division. The neurological assessment was performed by an AD specialist. All of the patients diagnosed using the criteria underwent a complete neuropsychiatric evaluation followed by a neuropsychological evaluation. Cognitive screening tests, a neuropsychological battery and a functional assessment were used for patient selection and group classification. The Mini-Mental State Examination (MMSE) was used as a screening tool.^10^ The Portuguese translation and scoring of the MMSE was used.^11^ Only those individuals with an MMSE score > 12 and who were undergoing treatment for AD with a therapeutic dose of acetylcholinesterase inhibitors (donepezil 5 mg, rivastigmine 9 mg or galantamine 8 mg) were selected. The subjects were also assigned a Clinical Dementia Rating (CDR).^12^ A CDR score of 0.5 or 1 defined mild dementia and CDR 2 moderate dementia. For the neuropsychological evaluation, patients were assessed using the protocol established by the Consortium to Establish a Registry for Alzheimer's Disease (CERAD),^13^ which evaluates attention, memory, recall, recognition, language, praxis, gnosia, and abstract thinking using the following tests: verbal fluency, naming, word list memory, constructive praxis, word list recall, word list recognition, praxis recall, and the trail-making test.

All individuals who met the inclusion criteria were administered the Boston Diagnostic Aphasia Examination (BDAE).^14^ This test was chosen because it provides a broad assessment of auditory/oral and written comprehension and of oral and written production. Only the tasks relating to language assessment were applied.

The following tasks were performed:

• Oral comprehension: word discrimination, body-part identification and complex ideational material.
• Speech tasks: automated sentences, repetition of words and repetition of high and low-probability sentences, oral reading of words, responsive naming, and visual confrontation naming.
• Reading comprehension: symbol discrimination, word recognition, oral spelling, word-picture matching, comprehension of sentences and paragraphs.
• Writing: serial writing, primer-level dictation, spelling to dictation, writing confrontation naming and narrative writing.

All patients were assessed by the same examiner through individual assessments performed in a quiet room. Sessions lasted for less than one hour.

Based on the results of the assessments, an analysis of the frequencies of the variables outlined above was conducted.

Statistical analysis – the Chi-Squared (χ^2^) (without Yates correction) test, or Fisher's exact test (when contingency tables displayed expected value < 5), was applied to compare categorical data.

Differences between means of continuous data were tested using Student's *t-*test for paired samples (*t*) (parametric), whereas the Wilcoxon Signed Ranks (Z) test was used for their corresponding non-parametric samples. Parametric results were displayed when the two had similar results, while non-parametric results were shown when divergence occurred.

Bonferroni correction for multiple comparisons was used. A p-value of < 0.002* was considered statistically significant and all tests were two-tailed. A 95% Confidence Interval (95% CI) was calculated for differences in means. Statistical analyses were carried out on a personal computer using the statistical package SPSS for Windows (version 11.5.1).

## Results

### General characteristics

No statistically significant difference was found between the Mild AD and Moderate AD groups with respect to age (70.2 [SD+9.6] vs. 74.45 [SD+8.7] years; 95% CI 10.6‒2.1; t(33) = -1.3; p = 0.183) or gender (42.9% vs. 57.1% for men; p = 0.317). The group with mild AD had significantly more years of education than the moderate AD group (9.79 [SD = 6.1] vs. 4.05 [SD + 2.6] years; 95% CI 2.5‒8.9; t(31) = 3.6; p = 0.001*). As expected, Moderate AD patients had significantly lower mean scores on the MMSE than Mild AD patients (25.53 [SD+1.2] vs. 17.65 [SD = 3.2]; 95% CI 6.0‒9.0; t(33) = 8.9; p < 0.001).

The descriptive analysis of mild and moderate AD patient results on the BDAE is presented in Table 1.

**Table 1:** Statistical data on mild and moderate stage AD groups.

	Mild AD				Moderate AD				NI[15]
BDAE tasks	Mean	SD	Minimum	Maximum	Mean	SD	Minimum	Maximum	Cut off score
ORAL COMPREHENSION									
Word discrimination	69.46	4.48	55.5	72.0	55.82	13.93	12.0	71.0	62
Body-part identification	18.25	1.15	16.0	20.0	15.57	2.65	9.0	19.5	16
Complex Ideational Material	7.78	1.47	5.0	10.0	6.26	1.24	4.0	8.0	8
SPEECH TASKS									
Automated Sequences	7.26	0.45	7.0	8.0	6.70	0.80	4.0	7.0	7
Repetition									
Words	9.80	0.56	8.0	10.0	9.15	1.22	6.0	10.0	9
High-probability phrases	7.40	0.91	5.0	8.0	6.40	1.69	2.0	8.0	7
Low-probability phrases	7.26	0.79	6.0	8.0	5.15	2.18	0.0	8.0	6
Naming									
Responsive	27.00	0.00	27.0	27.0	26.26	1.04	24.0	27.0	25
Visual Confrontation	105.0	9.26	84.0	114.0	85.75	15.54	54.0	110.0	102
Oral reading									
Words	29.73	1.03	26.0	30.0	24.94	5.73	13.0	30.0	27
READING COMPREHENSION									
Symbol discrimination	9.66	0.61	8.0	10.0	7.38	2.06	2.0	10.0	9
Word recognition	7.78	0.80	5.0	8.0	6.94	1.62	3.0	8.0	7
Oral spelling	4.78	2.42	1.0	8.0	2.00	1.79	0.0	5.0	3
Word-picture matching	9.64	0.49	9.0	10.0	6.81	2.94	0.0	10.0	7
Sentences and paragraphs	9.00	1.17	6.0	10.0	5.33	3.39	0.0	9.0	7
WRITING									
Mechanics									
Serial writing	43.71	3.17	35.0	47.0	32.56	10.05	8.0	45.0	36
Primer-level dictation	14.64	0.63	13.0	15.0	11.58	3.06	2.0	15.0	12
Spelling to dictation	8.92	0.95	7.0	10.0	5.64	3.31	0.0	9.0	6
Written confrontation naming	9.14	1.91	3.0	10.0	4.56	3.42	0.0	10.0	8
Narrative writing	10.57	2.06	6.0	12.0	5.53	4.30	0.0	11.0	3

NI, Neurotypical Individuals data from Radanovic and Mansur (2002)[15] study.

The cut-off scores presented in Table 1were suggested by the study of Radanovic, Mansur.^15^

The statistical analysis of the data comparing the performance of the two groups on the BDAE tasks is presented in Table 2.

**Table 2:** Comparison of mean scores of mild and moderate Alzheimer's disease patients on BDAE tasks.

BDAE tasks	95% CI (difference)	*t*	DF	p-value
Word discrimination	5.74‒21.53	3.5	32	0.001*
Body-part identification	1.13‒4.21	3.5	32	0.001*
Complex Ideational Material	0.55‒2.48	3.2	31	0.003*
Low-probability phrase repetition	0.91‒3.32	3.5	33	0.001*
Word Reading	1.71‒7.85	3.1	32	0.003*
Responsive naming	0.18‒1.28	2.7	32	0.010*
Visual confrontation naming	10.10‒28.52	4.2	33	0.000*
Symbol discrimination	1.14‒3.40	4.1	31	0.000*
Oral spelling	1.12‒4.44	3.4	26	0.002*
Word picture matching	1.19‒4.46	3.5	28	0.001*
Sentence and paragraph reading	1.70‒5.63	3.8	27	0.001*
Serial writing	5.40‒16.90	3.9	28	0.000*
Primer-level dictation	1.34‒4.76	3.6	29	0.001*
Spelling to dictation	1.32‒5.22	3.4	28	0.002*
Written confrontation naming	2.46‒6.69	4.4	28	0.000*
Written sentences	2.43‒7.64	3.9	27	0.000*

A statistically significant difference in patient performance was detected. The results of mild AD patients were superior to those of moderate AD patients for all BDAE tasks.

Non-parametric tests were needed for some tasks, such as automated sequences (U = 93.50; p = 0.007*), word repetition (U = 101.50; p = 0.049*), word recognition (U = 81.00; p = 0.031*) and repetition of high-probability phrases (U = 96.50; p = 0.059). The results revealed a non-statistically significant group difference only for the latter repetition task.

Analysis of the association between the groups for total score on the BDAE tasks revealed a statistically significant difference in scores (95% CI 47.10‒114.08; *t* = 5.0; DF = 21; p = 0.000*).

Linear regression analysis was performed to determine whether the total score on BDAE tasks was influenced by group (mild and moderate AD), independently of the variables sex, age, and education (Table 3).

**Table 3:** Results of multiple linear regression analyses for BDAE performance.

BDAE total score	ß	Standard Error	*t*	95% CI (difference)	p
Group	-0.73	21.53	-3.86	-128.87‒37.56	0.001*
Sex	-0.17	19.69	-1.03	-62.09‒21.40	0.317
Age	-0.02	0.93	-0.18	-2.15‒1.80	0.852
Education	0.05	2.18	0.28	-4.01‒5.23	0.782

## Discussion

As expected, the language performance of mild AD patients was superior to that of moderate AD patients. The decline in language performance correlated with the worsening of dementia syndrome, independently of sociodemographic variables. There was clear evidence that some linguistic tasks were more compromised than others. It seems that the more highly demanding is the linguistic task, the more compromised are the results obtained. In fact, some linguistic tasks involve more cooperation and involvement with cognitive systems than others,^4^ especially the interactions between short-term memory,^16^,^17^ that is typically affected in patients with AD, and language processing. Generally, there was a marked decline during the moderate stage, reflected by score differences on all BDAE tasks, except the high-probability phrase repetition task. Although some linguistic skills are more affected than others, that is, the pattern of impairment is heterogeneous the worsening, in terms of the ability that is compromised, is similar in both groups. The analysis of changes in processing during the disease course for the components assessed i.e., oral comprehension, oral production, reading, and writing is further discussed below.

Regarding the auditory/oral word discrimination and body-part identification tasks, the performance of mild AD patients was superior to that of moderate AD patients, indicating impaired semantic processing of words. On the complex ideational material task, involving text and sentence comprehension, patients with moderate AD again exhibited worse performance compared to individuals at a mild stage of the disease. However, despite the clinical worsening, manifested by a higher number of errors, impaired performance on this task is already evident at the mild stage of the disease.^18^ The phrase and text comprehension involved in this subtest demands concomitant processing of syntactic and semantic elements. Thus, besides changes in the language processing of information, working memory is required for the temporary storage of language information during syntactic-semantic processing.^17^ The demand placed on semantic and working memories may be greater or lower depending on the complexity of the sentences.^19^ Analysed together, a major difference between the 2 groups for comprehension tasks was observed in sentence and paragraph comprehension.

Regarding oral production tasks, automated sequences require individuals to recite ultra-learned verbal content, an extremely simple skill that occurs independently of the association with meaning.^20^ The poorer performance of individuals with moderate AD relative to those with mild AD is due to the latter group's need for prompts to produce the series and number of items within each sequence. The need for prompts may have arisen from difficulty encountered by some moderate-stage patients in initiating the utterance due to loss of initiative or lack of understanding on exactly what they are supposed to do or to access semantic information, e.g., doubts over what days of the week or months of the year are, leading to points lost. The fact that some patients break off the sequence before reaching the end might be due to the absence of reverberation of the instruction until full execution.

With regard to oral repetition tasks, the BDAE presents items divided into 3 categories. When using the simplest stimuli (words), moderate AD patients had worse performance than mild AD patients. The same performance pattern was seen for low-probability phrases. By contrast, for high-probability phrase repetition, both mild and moderate AD patients showed similar performance. Repetition of single words can be performed directly by the phonological system, without recruiting the semantic system. In this case, however, the demands are higher on this system, which appears to be more preserved in the early stages of AD.^21^ Nevertheless, the task can also be executed by lexical-semantic or lexical-phonological decoding. Greater impairment of either of these processes can explain the worse performance of moderate AD patients on the word repetition task. Regarding low-probability phrase repetition, greater demand is placed on phonological processing and working memory,^22^ both of which are increasingly impaired with AD progression. Although AD patients tend to encounter greater difficulty when dealing with more complex stimuli, in the present study, performance differed only for familiarity with the stimuli. For high-probability repetition, the authors hypothesized that moderate AD patients may have benefited from concomitant phonological and syntactic-semantic processing. Moreover, given these tasks involve motor planning, the occurrence of phonetic errors cannot be ruled out, because, although less common in AD,^23^ this class of error worsens with disease progression.^24^

In the confrontation naming task, the items are presented on cards and the patient has to name them as directed by the examiner. The moderate AD patients assessed in the present study had worse performance compared to subjects with mild AD. In fact, in the moderate stage of AD, lexical-semantic impairment worsens, not only in terms of the number of errors but also in relation to error type where, besides an increase in anomia, verbal paraphasia also begins to emerge.^25^ Errors identified on this task might be related to the complexity present in low-probability stimuli or perhaps stem from perceptual, visual, or attentional deficits that can affect naming activities.

On the naming test using semantic cues, although mild AD patients produced fewer errors, moderate AD patients performed better on this task than on visual confrontation naming. A previous study^3^ found differences between moderate AD patients and healthy subjects on a naming task in which semantic cues were presented, probably because the patients benefited from semantic cues indicating object function. The authors argued that the description of use/function improves lexical access for these patients. Also, the semantic categories explored in this task include content considered routine and common, likely facilitating recall.

On the reading comprehension assessment, performance on all tasks was impaired in individuals with moderate AD relative to those with mild AD.

With regard to word and symbol processing, temporary storage of information is necessary to allow subsequent selection of the form corresponding to the stimulus displayed in the graphemic buffer. This temporary storage, which occurs via working memory, can be impaired. As a result, the matching of the form indicated by the words presented and the corresponding form in another allographic representation is either not possible or fails to occur correctly. Other hypotheses may also explain this difficulty, such as changes in visual perception or a failure in the graphemic buffer. Considering the graphemic buffer's role in maintaining the order and identity of abstract letter identities, it is assumed that errors at this level reflect a loss of information about these graphemes.^26^ Serial position effects have been argued to reflect the presence of different components of orthographic working memory.^24^

With regard to oral spelling comprehension, it has been argued that the orthographic buffer is also required. In this instance, orthographic information pertaining, for example, to letter identity and order, is stored temporarily while output procedures and lexical matching are completed. In the case of oral spelling, this process consists of grapheme-letter name conversion that relies on the orthographic buffer.^27^

In Brazil, the use of oral spelling in school is unusual, possibly contributing to the poor performance of AD patients on this task, although statistical group differences were evident.

The poorer performance of moderate AD patients on the word-picture matching task might be explained by the deterioration in reading with subsequent semantic access difficulty.^28^ Reading may take place via different routes, such as lexical or phonological. However, based on results from the word reading assessment, it can be theorized that moderate-stage AD patients encounter difficulties with tasks involving the phonological and lexical buffers, as well as with accessing routes to these subsystems. Nevertheless, the relative preservation of reading words aloud suggests the occurrence of non-semantic translexical reading. This phenomenon has been reported previously in several studies,^3^,^28^ showing that the individual reads by transcoding the graphic to the oral buffer, giving the false impression of preserved reading when, in fact, there is no comprehension, because no access to semantic knowledge takes place.

Group differences were also evident for sentence and paragraph reading. Reading comprehension problems are a common complaint in AD patients at the early stages of the disease, a process that relies on linguistic components and specific aspects of cognitive processing. On the sentence and paragraph reading subtest, increasingly complex phrases are presented that require patients to critically analyze the content in order to adequately complement the phrases. In addition, the test offers multiple choices which require more working memory and attention, processes naturally involved in reading texts and commonly impaired in individuals with AD.

On the writing assessment, performance on all tasks was impaired, with mild AD patients performing better on the task than individuals with moderate AD.

Writing disorders are an early manifestation of Alzheimer's Disease (AD) and may often be more severe than oral language difficulties,^29^ although agraphia conditions can be highly heterogeneous.^3^,^30^

On the serial-writing task, in which patients write a numerical (1 to 21) and alphabetical sequence, the low performance of moderate AD patients might be due to a failure in working memory involved in the reverberation and serializing/ordering of the elements. A previous study found that AD patients had less automated movements and lower writing velocity,^31^ factors that might also impact the task of writing a serial order.

On the primary-level dictation and spelling to dictation writing tasks, the mild AD group performed better than the moderate group, a finding explained by the preservation of the phonological route, enabling mild AD patients to match phonemes and graphemes, although this route is not necessarily preserved in all cases of AD. Sentence writing was also impaired. As observed previously, AD patients produce shorter writing, which may also include intrusions and misspellings.^31^ Also, graphemic and grapho-motor^32^ deficits can co-occur in all written tasks.

One final clinical observation is that the overall BDAE results in patients with moderate AD seem to show that patients scoring over 17 points on the MMSE, despite being at moderate stage, displayed more similar language performance to mild-stage patients. Then, this observation suggests that it is possible that the language profile of these patients is not the same through the moderate stage and language deterioration is a continuum that runs in parallel with cognitive worsening.

## Conclusion

The results of the different BDAE subtests revealed that a decline in language performance correlated with the worsening of dementia syndrome, independently of sociodemographic variables. Although the study results showed major changes in the moderate stage, there were linguistic tasks more preserved than others. More studies are necessary to find out possible correlations between language performance and other cognitive functions decline. The assessment of language can shed light on the linguistic abilities affected in these individuals and help guide stimulation strategies to maintain communication during the disease course. Implementing such interventions to aid communication remains challenging for many health professionals in the field.^4^

## Declaration of competing interest

The authors declare no conflicts of interest.
